# Health parameters for wild Carnaby's cockatoo (*Zanda latirostris*) nestlings in Western Australia: results of a long-term study

**DOI:** 10.1093/conphys/coae005

**Published:** 2024-02-20

**Authors:** Anna T Le Souëf, Mieghan Bruce, Amanda Barbosa, Jill M Shephard, Peter R Mawson, Rick Dawson, Denis A Saunders, Kristin S Warren

**Affiliations:** School of Veterinary Medicine, College of Environmental and Life Sciences, Murdoch University, South Street, Murdoch, Western Australia, 6150, Australia; Department of Biodiversity, Conservation and Attractions, Perth Zoo, Locked Bag 104, Bentley DC, Western Australia, 6983, Australia; Centre for Biosecurity and One Health, Harry Butler Institute, Murdoch University, South Street, Murdoch, Western Australia, 6150, Australia; Centre for Biosecurity and One Health, Harry Butler Institute, Murdoch University, South Street, Murdoch, Western Australia, 6150, Australia; Centre for Terrestrial Ecosystem Science and Sustainability, Harry Butler Institute, Murdoch University, South Street, Murdoch, Western Australia, 6150, Australia; Department of Biodiversity, Conservation and Attractions, Perth Zoo, Locked Bag 104, Bentley DC, Western Australia, 6983, Australia; Waikiki, Western Australia, 6169, Australia; Weetangera, Australian Capital Territory, 2614, Australia; Centre for Terrestrial Ecosystem Science and Sustainability, Harry Butler Institute, Murdoch University, South Street, Murdoch, Western Australia, 6150, Australia

**Keywords:** Australia, avian health, avian polyomavirus, baseline health, beak and feather disease virus, black cockatoo, Carnaby’s cockatoo, *Chlamydia*, reference intervals, wildlife health

## Abstract

The collection of baseline health data is an essential component of an endangered species conservation program. As for many wildlife species, there are minimal health data available for wild populations of the endangered Carnaby’s cockatoo (*Zanda latirostris*). In this study, 426 wild Carnaby’s cockatoo nestlings were sampled from nine breeding sites throughout the range of the species over an 11-year period. In addition to a physical examination, samples were collected to test for hematologic and biochemical parameters, psittacine beak and feather disease virus (BFDV), avian polyomavirus (APV), psittacine adenovirus, psittacine herpesvirus, *Chlamydia*, disease serology and endoparasites. Environmental sampling was performed to screen for BFDV and APV in nest hollows. Descriptive health data are presented for nestlings of this species, with BFDV, APV and *Chlamydia* infections reported. Reference intervals for hematologic and biochemical parameters are presented in three age groups, and factors affecting blood analytes and body condition index are discussed. This longitudinal dataset provides insights into health parameters for Carnaby’s cockatoo nestlings and a reference for future monitoring of breeding populations.

## Introduction

The collection of baseline biological data from wildlife in natural habitats is essential to understand disease status and provides a benchmark with which future information can be compared (Smith *et al.,* 2009; Mallory *et al.,* 2010; Parker and Deem, 2012). As part of developing a better understanding of the ecological processes occurring within populations, the measurement of blood parameters has been increasingly used (Minias, 2015) and forms a useful part of health monitoring programs for wildlife. In Australia, health assessment studies have been reported for many species of wildlife, but few exist for wild psittacine populations, including Carnaby’s cockatoo (formerly *Calyptorhynchus latirostris* and now *Zanda latirostris*).

Carnaby’s cockatoos are endemic to southwest Western Australia, breeding mainly in eucalypt woodlands in the semiarid and subhumid interior (Saunders, 1979b). They generally lay two eggs approximately one week apart, but will usually fledge only one nestling depending on the experience and age of the female, and the extent to which surrounding vegetation has been cleared (Saunders *et al.,* 2014). Threats to wild Carnaby’s cockatoo populations include habitat fragmentation and destruction, competition with other species for nest hollows, poaching for the illegal pet trade, vehicle strike, disease and climate change (Saunders, 1990; Mawson and Johnstone, 1997; Saunders and Ingram, 1998; Saunders *et al.,* 2011; Le Souëf *et al.,* 2015; Le Souëf *et al.,* 2020). The cockatoo is a highly visible and charismatic species, protected by federal law under the Environment Protection and Biodiversity Conservation Act 1999, but continues to show population declines (Department of Climate Change, Energy, the Environment and Water, 2023). In the Australian national Carnaby’s Cockatoo Recovery Plan, disease is listed as a known threat, and recovery actions include ‘(to) conduct longitudinal studies of the epidemiology and clinical significance of beak and feather disease virus (BFDV) and avian polyomavirus (APV) infections in wild populations’ (Department of Parks and Wildlife, 2013). With ongoing anthropogenic changes to the environment, including habitat clearance, landscape modification and climate change, it is important that Carnaby’s cockatoo populations are closely monitored, and that their baseline health is well understood.

Breeding sites of Carnaby’s cockatoos in Western Australia have been monitored on an ongoing basis for hollow occupancy, breeding attempts and fledgling success for decades (Saunders, 1979a; Saunders, 1982; Saunders, 1986; Saunders and Ingram, 1998; Saunders *et al.,* 2014; Saunders and Dawson, 2018), providing the opportunity for sample collection from both nestlings and from their nest hollows during nest monitoring trips. Several disease pathogens were selected for testing, including BFDV, APV, psittacine adenovirus (PAdV), psittacine herpesvirus (PHV) and *Chlamydia psittaci* on the basis of their identification as significant pathogens threatening psittacine populations globally (Wellehan *et al.,* 2016; Stokes *et al.,* 2020; Vaz *et al.,* 2020). Indirect transmission of BFDV through contaminated nest hollows may be an important infection source (Martens *et al.,* 2020a), so in addition to the testing of nestlings for disease pathogens, nest substrate samples were tested for BFDV and APV which may also be found in the environment. Along with disease prevalence studies, clinical pathology is an informative way of assessing health status in wild bird populations (Artacho *et al.,* 2007). Therefore, as well as samples for disease screening, blood samples were also collected during nest survey trips to determine blood reference intervals for hematology and biochemistry in Carnaby’s cockatoo nestlings.

## Materials and Methods

### Sampling

**Figure 1:**
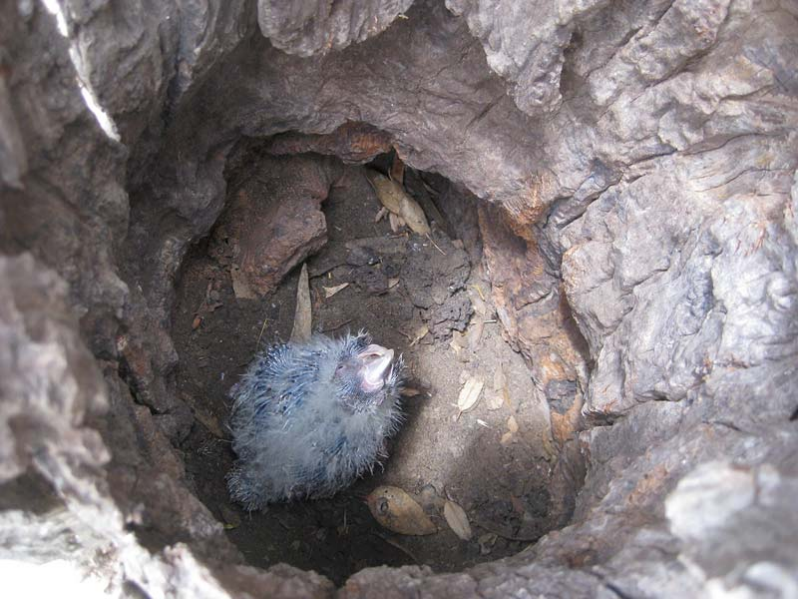
Carnaby’s cockatoo (*Zanda latirostris*) nestling in a natural nest hollow.

**Figure 2:**
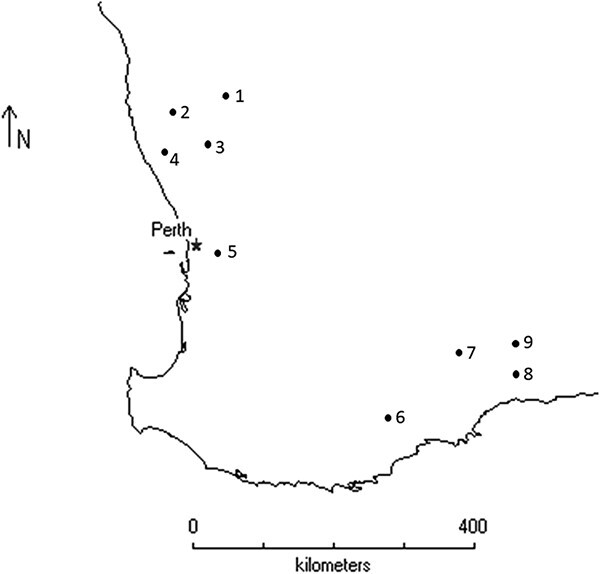
Location of study sites for determination of baseline health data in Carnaby’s cockatoo (*Zanda latirostris*) nestlings in south-west Western Australia (1 = Coorow, 2 = Coomallo Creek, 3 = Moora, 4 = Cataby, 5 = Glen Forrest, 6 = Borden, 7 = Newdegate, 8 = Cocanaraup, 9 = Lake King).

Samples were collected from 426 Carnaby’s cockatoo nestlings (Figure 1) along with the substrate of 171 nest hollows at nine sites across the breeding range in southwest Western Australia (Figure 2), annually between October and December from 2010 to 2015, 2020 and 2021. The sites were on private agricultural land, roadsides and timber reserves. Nesting hollows were in mature salmon gum (*Eucalyptus salmonophloia*) or wandoo (*E. wandoo*) or artificial hollows attached to mature trees (Saunders *et al.,* 2020). Nestlings were removed from nest hollows, held briefly under manual restraint and sampled shortly after removal. A cloth was placed over the nestling’s head to reduce stress during handling. Nestlings were examined for external health abnormalities, weighed (g) and the length of the folded left wing (mm) recorded. Nestlings were aged on the length of the folded left wing following the method following the method of Saunders (1986), and each nestling was assigned a body condition index following the method of Saunders *et al.* (2019), which calculates the percentage deviation of body mass from an established benchmark body mass for age.

Blood was collected from the medial metatarsal vein using a pre-heparinized needle and syringe into a lithium heparin tube, and fresh blood smears were made on glass slides and air dried. A temporary bandage using a calcium alginate dressing and paper tape was useful in aiding coagulation following phlebotomy, thus reducing handling time. A combined swab was taken of the conjunctivae, choanae and cloaca using a sterile dry swab. Feces and nest hollow substrate were also collected. All samples were chilled in the field in a portable cooler, then blood tubes were stored at −4°C, and swabs, blood spots and nest hollow substrate samples were frozen at −20°C. Blood and fecal samples were packed in coolers with ice for transportation from the field and were analysed within 48 hours of collection.

### Blood and fecal analysis

Hematologic analysis was performed on a Cell-Dyn 3500 automated hematology analyzer (Abbott Diagnostics, Abbott Park, Chicago, IL, USA), run in resistant cell mode. Red blood cell (RBC) count, white blood cell (WBC) count, hemoglobin (Hb), mean corpuscular volume (MCV), mean corpuscular hemoglobin (MCH) and mean corpuscular hemoglobin concentration (MCHC) were measured. Packed cell volume (PCV) was measured using standard hematocrit methods after centrifugation in a Hettich Zentrifuge Haematocrit 20 (Hettich, Tuttlingen, Germany) for 3 minutes at 7500 rpm. MCV, MCHC and MCH were calculated using standard formulas from RBC, PCV and Hb concentrations (Ravi Sarma, 1990). Smears were stained with Leishman's stain for a manual differential leukocyte count and hemoparasite screen. Two hundred leukocytes per smear were counted and characterized as heterophils, monocytes, lymphocytes, eosinophils or basophils based on morphological characteristics. Heterophil to lymphocyte ratios were not included due to the limitations in interpretation associated with measurement uncertainty (Clark *et al.,* 2022). Biochemical analyses were performed on serum using an Olympus AU 400 Automated Chemistry Analyzer (Olympus Optical Company, Tokyo, Japan).

Fecal samples were screened for parasites by microscopic examination of wet mounts, and zinc sulphate concentration for detection of cysts or ova. Blood was tested for *C. psittaci* serology using the ImmunoComb® Avian *C. psittaci* solid-phase ELISA Antibody Test Kit (Biogal Galed Labs, Israel) (Biogal, 2013). DNA sexing was performed at commercial laboratories using blood on filter paper.

### Molecular testing

From 2010 to 2015, swabs, feathers, blood spots and nest substrate were tested at the Veterinary Diagnostic Laboratory at Charles Sturt University, Wagga Wagga, Australia using PCR for detection of BFDV, APV, PAdV and *Chlamydia*, and hemagglutination and hemagglutination inhibition testing for BFDV using previously described protocols (Raidal *et al.,* 1993; Ypelaar *et al.,* 1999; Wellehan *et al.,* 2004; Khalesi *et al.,* 2005; Robertson *et al.,* 2009). In 2020 and 2021, swabs, blood spots and nest hollow substrate swabs were tested at the Veterinary Pathology Diagnostic Services at the University of Sydney, Australia using PCR for BFDV, APV, PHV and *Chlamydia* using the AusDiagnostic Avian Kit®. For DNA extraction from nest hollow substrate samples, 2 g of material was placed into a 50-ml centrifuge tube, to which 5 ml of phosphate buffered saline was added, the tube vortexed and boiled for 30 minutes. The sample was then centrifuged at 3500 *g* for 10 min, and the supernatant syringed through 0.22-μm filters into 5-ml sterile tubes. The extraction was continued with the Qiagen DNA Stool Kit (Qiagen, Hilden, Germany). To increase the sensitivity of the testing, in 2020 and 2021, sampling of nest hollow substrate was undertaken by inserting a swab into the bag containing the material, such that it contacted most surfaces of the nest material; the swab was then subjected to DNA extraction as previously described.

### Phylogenetic analysis

For BFDV, primers within open reading frame 1 (ORF1) region of the BFDV genome were used to amplify a 700-bp fragment. The PCR product from one nestling's blood spot sample was sequenced following purification at Macrogen, Inc (Seoul, Korea). The partial BFDV nucleotide sequence obtained was aligned with BFDV genome sequences retrieved from GenBank, representing known genotypic clades of BFVD previously described in Psittaciformes in Australia and overseas. Sequences were aligned using MAFFT v7.388 (Katoh *et al.,* 2002; Katoh and Standley, 2013) in Geneious Prime 2022.2.2 (https://www.geneious.com). The alignment was then trimmed, and a preliminary phylogenetic tree generated using Geneious FastTree (Price *et al.,* 2009). Due to the relatively short length of the alignment (which hindered reliable comparisons between distantly related genotypes), a subset of partial sequences most closely related to the novel genotype was selected for further analysis. The subsequent alignment was imported into MEGA 11 (Tamura *et al.,* 2021) and the most appropriate nucleotide substitution model was selected using the dedicated function. A maximum likelihood phylogenetic tree was constructed using Kimura 2-parameter nucleotide substitution model (Kimura, 1980), with rates among sites gamma distributed with invariant sites (G + I). Reliability for internal branch was assessed using the bootstrapping method (500 bootstrap replicates) and support values (>60%) indicated the left of each node. Pairwise genetic distances were calculated using the maximum composite likelihood method in MEGA 11.

### Statistical methods

Blood samples collected between 2010 and 2020 were used to determine blood reference intervals (this part of the analysis was completed before the blood samples from 2021 were collected). The statistical package SPSS version 24 (SPSS Inc., Chicago, IL, USA) was used for statistical analysis, and MedCalc Statistical Software version 20.009 (MedCalc Software Ltd, Ostend, Belgium) was used for calculation of reference intervals in accordance with American Society for Veterinary Clinical Pathology guidelines (Friedrichs *et al.,* 2012). For the development of blood reference intervals, nestlings were removed from the dataset if there was external evidence of poor health or injury. Statistical outliers were detected using the Tukey’s test and removed from the dataset. Sex was considered as a factor for partitioning, however when guidelines were followed for partitioning (Lahti *et al.,* 2002; Lahti *et al.,* 2004), the method appeared too sensitive, resulting in partitioning deemed impractical for clinical use, so both sexes were combined in the reference intervals. Distribution of reference data was assessed by examined histograms and confirmed with a goodness-of-fit (Shapiro–Wilk) test with a *p* value of 0.05. If at least 120 reference values were available, non-parametric methods were used to determine the central 95% of reference values, along with 90% confidence intervals around these limits. When fewer than 120 reference values were available, robust methods were used to determine reference intervals. In these cases, if data were not normally distributed, they were first transformed using Box-Cox or log transformation, and back-transformed to generate reference intervals. In addition to the reference intervals, mean, standard deviation, median and minimum/maximum values were calculated. Methods for estimating reference intervals varying with continuous age are statistically complex and not widely agreed upon (Hoq et al., 2020). Further, as a reference, they are not as practically useful as grouped reference intervals. Therefore, in this study, the data have been presented in three age groups (≤5 weeks, 6–8 weeks and ≥ 9 weeks old) after considering the amount of variation between the age groups and sample sizes (i.e. only parameters that contained sufficient numbers in each age group were categorized into age groups).

For the analysis of factors affecting blood analytes, frequency histograms were generated for each blood parameter and then distributions were assessed using the Shapiro–Wilk test with a *p* value of 0.05. The effect of predictors for the following blood analytes was examined: PCV, WBC, absolute heterophil count, absolute lymphocyte count, absolute eosinophil count, total protein, calcium, CK, AST, uric acid, albumin and globulin. First, categorical independent variables were assessed using t-tests and ANOVA for groups with a normal distribution, and Mann–Whitney U tests and Kruskal–Wallis tests for groups that showed nonparametric distributions. The relationship with each blood analyte was assessed using Pearson's correlation tests. Linear regression models were constructed using the backward stepwise elimination method to determine possible predictors of blood analyte measurements out of the following candidate variables: body condition index, year, location (categorized into three groups: Coomallo Creek, Coorow/Moora and the remaining southern sites); age; sex; hollow type (natural or artificial); sibling status (whether a sibling was present in the hollow); ambient temperature; *Chlamydia* PCR positivity; time of day (at which sampling was undertaken); and days after commencement of egg laying (i.e. the number of days the nestling hatched after egg laying commenced in the breeding area). Predictor variables were chosen that could be measured reliably and that were considered to have a realistic potential relationship with blood analytes. Independent variables included in the multiple regression models were those identified in the preliminary correlation analyses to have at least moderate correlation (*P* < 0.25) with the blood analyte. Categorical variables were dummy-coded and continuous variables were centred. Interaction terms were computed, and entered into each model and plotted to check for interaction effects. Analyses were performed to check that there was no violation of the assumptions (normality and linearity) of a multiple linear regression. Another linear regression model was also constructed with body condition index as a dependent variable, with independent variables included as in the blood analyte models. Data from 38 sibling sets were also analysed for potential differences in blood analytes and body condition indices between younger and older siblings using t tests or Mann–Whitney U tests, depending on normality of data.

### Ethical declarations

The capture and handling of the Carnaby’s cockatoo nestlings was conducted under Western Australian Department of Environment and Conservation (DEC) scientific purposes licences SC000357, SC000920, SC001230, Australian Bird and Bat Banding Authority 1862 and DEC Animal Ethics Committee approvals DEC AEC 11/2005, 32/2008, 30/2011, 23/2014, 21/2017 and Department of Biodiversity, Conservation and Attractions AEC approval 2020-10C held by PRM and Section 40 Authorisations TFA 2019-0111-1, 2019-0111-2 and 2019-0111-3 held by RD. The veterinary sampling was conducted with the Murdoch University Animal Ethics Committee approvals W2377/10 and RW3232/20.

## Results

A low proportion of Carnaby’s cockatoo nestlings were PCR positive for BFDV (7/419 or 1.7%; 95% CI, 0.8–3.4) and APV (12/416 or 2.9%; 95% CI, 1.7–5.0), while the *Chlamydia* PCR prevalence was 9.8% (38/388 or 95% CI, 7.2–13.2) (Table 2). Three nestlings had concurrent BFDV and APV infections, and two nestlings had concurrent APV and *Chlamydia* infections. None of the cockatoos tested positive on PCR for PHV and PAdV. There were no overt clinical signs of ill health in any of the infected nestlings.

**Figure 3:**
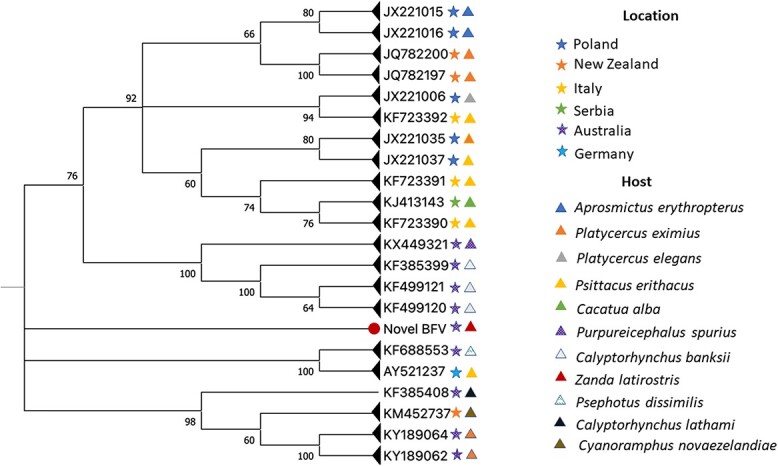
Phylogenetic relationships of the novel BFDV genotype (indicated by a circle next to its name) with other BFDV isolates retrieved from Genbank, based on partial sequences (703 bp). Bootstrap values (>60%) based on 500 replicates are indicated at the left of each supported node.

Phylogenetic analysis revealed that the BFDV sequence recovered from one nestling blood spot sample in this study (taken in Newdegate in 2021) represented a novel genotype situated in a separate branch with low bootstrap support (<60%) (Figure 3). This variant shares a common ancestor with isolates from a range of Psittaciforme species from Australia, New Zealand and Europe (Figure 3).

**Table 1:** Descriptive data for Carnaby’s cockatoo (*Zanda latirostris*) nestlings (n = 339) included in the development of hematologic and biochemical blood reference intervals

		Cohort (n)	Age ≤ 35 days	Age 36–56 days	Age ≥ 57 days
Sex					
	Male	167	33	113	21
	Female	172	37	103	30
Location					
	Coomallo Creek	222	50	134	38
	Southern	86	16	61	9
	Coorow/Moora	30	6	20	4
Nest type					
	Natural	203	38	131	34
	Artificial	137	34	86	17
Sibling					
	Absent	274	55	181	38
	Present	66	17	36	13

The overall nucleotide identity of the novel BFDV isolate ranged from 91.7% to 95% compared to the BFDV sequences available in GenBank (partial sequences). The novel genotype was most closely related (95% pairwise identity) to genotypic variants recorded in red-winged parrots (*Aprosmictus erythropterus*) and African grey parrots (*Psittacus erithacus*) from Poland (GenBank Accession Numbers JX 221015 and JX221037, respectively), and African grey parrots from Poland (GenBank Accession KF723390). In Australia, the most closely related BFDV genotype (94.7% pairwise identity) was previously isolated from red-tailed black cockatoos (*Calyptorhynchus banksii*) in Western Australia (GenBank Accession KF385399). The newly reported BFDV partial DNA sequence has been deposited in GenBank under accession number OQ659411.

All nestlings for which hemagglutination tests were performed on feathers (n = 347) had a negative result. All samples from nestlings for which BFDV serology (hemagglutination inhibition) (n = 353) and *C. psittaci* serology (n = 421) were performed were negative for antibodies. No endoparasites were seen in any of the 120 fecal samples submitted for endoparasite screening, and no hemoparasites were seen in any of the 385 blood smears examined. A single tick, from the genus *Amblyomma*, was recovered from the face of a nestling in an artificial hollow in Borden.

Of 171 nest hollow substrates that were sampled for BFDV PCR, two tested positive. The first, sampled in 2011, was from an artificial hollow at Coorow. It did not contain a nestling in the year of sampling (or in subsequent years). The second was from the nest hollow substrate of the Newdegate nestling that tested PCR positive for BFDV in 2021. This nest was sampled in July 2022, 223 days following the sampling of the nestling. The nestling in 2020 that tested positive for BFDV had a concurrent nest sample taken at the same time, which tested negative on PCR. Of the 140 nest samples tested for APV, none showed a positive PCR reaction.

Eight nestlings were removed from the blood reference interval dataset due to an external appearance of poor health (including beak wounds, very weak or lethargic demeanour, poor body condition, abnormal ocular discharge and abnormal respiration). One of these eight nestlings was euthanized due to a tibiotarsal fracture malunion. Necropsy examination revealed sunflower seeds in its crop, showing that its parents were feeding on an artificial diet, and the nestling was diagnosed with metabolic bone disease. Another nestling from the excluded group failed to fledge and was taken into care but died despite supportive treatment. The remaining six nestlings were left in parental care; all were likely to have fledged (as their hollows were later checked and were empty), and no further diagnoses were made. Thirty-five nestlings that were PCR-positive for *Chlamydia* were also removed from the blood reference interval dataset; although they showed no overt clinical signs of disease, there was a significant negative statistical association with PCV (*p* < 0.001) and body condition index (*p =* 0.002) in these nestlings.

Blood measurements from three nestlings were also removed entirely from the dataset for the blood reference intervals following identification of multiple significant outliers of blood parameters (one due to severe hemolysis). Additionally, two biochemical analytes, glucose and potassium, were removed entirely from the dataset for all nestlings, due to the time delay between blood collection and analysis that typically cause pre-analytical error in these particular analytes (Lumeij, 2008), resulting in low clinical usefulness. Data from all nestlings removed from the reference interval dataset were included in analyses of factors affecting blood analytes and body condition index.

Descriptive data for the blood reference interval dataset (n = 339) are presented in Table 1. Nestlings ranged in age from 14 to 78 days (mean age, 48 days). The ratio of females to males was almost equal.

Combined hematologic and biochemical reference interval data for nestlings of all ages (n = 374) are presented in Tables 3 and  4. Reference interval data for nestlings partitioned into the three age groups are presented in Table 5. Native Gaussian distributions were identified only for packed cell volume and chloride (and for globulin and calcium in the youngest age group). Other blood analytes showed a non-parametric distribution. The distribution of each parameter (with associated *p* value) and the method used to determine the reference intervals are provided in Tables 3-5.

The assumptions of a multiple linear regression were met for each model that was constructed to determine predictors of each blood analyte. The results of the multiple regression tests are presented in Table 6 for PCV and AST as dependent variables (the remaining models, which had less correlation between the predictor variables and the analyte, are available in the Supplementary Material). For albumin and calcium, no variables were left in the model after multiple regression. For body condition index as a dependent variable, predictor variables were found to be age (*p =* 0.002), days after commencement of egg laying (*p =* 0.001) and *Chlamydia* status (*p =* 0.002).

There were no differences between older and younger siblings for the analytes tested: PCV; WBC; heterophil percentage; lymphocyte percentage; monocyte percentage; H/L ratio; total protein; AST; UA and globulins; or for body condition indices. There was a significant difference in CK levels between the siblings (*p =* 0.028) with younger siblings having significantly higher CK levels (mean 1876 U/L) compared with older siblings (mean 1312 U/L).

## Discussion

There are significant challenges to studying the health of wild cockatoos, including the safe capture of adult birds and the attainment of sufficient sample sizes. Carnaby's cockatoo nestlings are relatively safely accessible and can be sampled consciously in a minimally invasive manner. During this study, nestlings were out of the nest hollow for an average of 14 minutes while they were weighed, measured, leg banded and had the samples taken. Monitoring the health of Carnaby's cockatoo nestlings is therefore a viable way of gathering data on the health status of breeding populations.

There appears to be current stability in the general health of the nine Carnaby’s cockatoo nestling populations surveyed. Infections with BFDV, APV and *Chlamydia* were detected without evidence of clinical disease. However, increasing pressures on Carnaby’s cockatoo populations associated with degradation and reduction in size of habitat, and other threatening processes including climate change, may increase the risk of future disease outbreaks. For example, host-switching of beak and feather disease virus in Australia may be enhanced by greater competition for nest hollows among avian hosts, as well as increased viral persistence in an arid environment (Sarker *et al.,* 2014b). Reference values from our study provide a baseline to monitor impacts of anthropogenic and environmental change on breeding populations of Carnaby's cockatoos in the future.

**Table 2:** Results of pathogen screening in wild Carnaby’s cockatoo nestlings in Western Australia per year tested, for 2010–2015, and 2020–2021. For each pathogen, the number of nestlings that tested PCR positive is provided, with the total number of cockatoos tested shown in parentheses

	2010	2011	2012	2013	2014	2015	2020	2021
BFDV	5 (51)	0 (55)	0 (64)	0 (45)	0 (55)	0 (83)	1 (48)	1 (19)
APV	4 (51)	0 (58)	2 (64)	0 (45)	0 (55)	0 (83)	0 (48)	0 (19)
*Chlamydia*	0 (50)	11 (58)	33 (64)	0 (45)	1 (55)	0 (83)	0 (48)	0 (19)
PAdV	0 (50)	0 (58)	0 (64)	0 (45)	0 (55)	0 (83)	-	-
PHV	-	-	-	-	-	-	0 (48)	0 (19)

**Table 3:** Combined hematologic reference intervals for wild Carnaby’s cockatoo (*Zanda latirostris*) nestlings in Western Australia

Analyte	SI Units	n	Mean	SD	Median	Min	Max	p-value^a^	Distribution^a^	Method^c^	LRL of RI^b^	URL of RI^b^	CI 90% of LRL	CI 90% of URL
PCV	L/L	316	0.40	0.08	0.40	0.20	0.62	0.34	G	NP	0.24	0.56	0.22–0.26	0.54–0.58
RBC conc.	10^12^/L	84	2.9	-	2.9	2.1	4.0	0.0	NG	RT	2.1	3.9	2.0–2.2	3.7–4.1
Hemoglobin	g/L	83	126	-	123	90	195	0.03	NG	RT	93	165	89–98	157–174
MCV	fL	84	155	11	157	106	191	0.00	NG	R	134	178	128–140	173–184
MCHC	g/L	84	282	29	276	212	407	0.00	NG	R	215	332	201–232	316–347
MCH	pg	84	44	4	43	36	63	0.00	NG	R	34	51	32–37	49–54
WBC conc.	10^9^/L	373	14.9	6.7	14.3	1.5	45.0	0.00	NG	NP	4.5	31.1	3.6–5.4	26.4–36.1
Heterophil	%	374	53	15	54	11	94	0.01	NG	NP	22	80	20–25	77–82
Heterophil	10^9^/L	373	8.11	4.62	7.24	0.53	35.60	0.00	NG	NP	1.30	18.77	1.17–1.89	16.53–22.38
Lymphocyte	%	374	40	15	38	0	80	0.01	NG	NP	14	70	13–17	67–72
Lymphocyte	10^9^/L	373	5.69	3.01	5.22	0.00	16.40	0.00	NG	NP	1.32	13.10	0.99–1.59	11.68–14.49
Monocyte	%	374	3	4	2	0	22	0.00	NG	NP	0	16	0–0	13–18
Monocyte	10^9^/L	374	0.51	0.76	0.24	0.00	4.66	0.00	NG	NP	0.00	3.35	0–0	2.3–3.67
Eosinophil	%	374	2	2	1	0	11	0.00	NG	NP	0	7	0–0	6–9
Eosinophil	10^9^/L	373	0.22	0.30	0.14	0.00	2.09	0.00	NG	NP	0.00	0.99	0–0	0.84–1.32
Basophil	%	374	1	2	1	0	15	0.00	NG	NP	0	7	0–0	5–8
Basophil	10^9^/L	373	0.22	0.36	0.13	0.00	4.50	0.00	NG	NP	0.00	0.95	0–0	0.75–1.3

RI, reference interval; LRL, lower reference limit; URL, upper reference limit; PCV, packed cell volume; MCHC, mean cell hemoglobin concentration; WBC, white blood cell; conc., concentration; TS, total solids; TP, total protein; H:L ratio, heterophil to lymphocyte ratio

a ^a^Statistical method for establishing RI: P, parametric; NP, nonparametric; R, robust; T, transformed

b ^b^Test used for distribution and p-value threshold for interpretation. G, Gaussian; NG, non-Gaussian

**Table 4:** Combined blood biochemical reference intervals for wild Carnaby’s cockatoo (*Zanda latirostris*) nestlings in Western Australia

Analyte	SI Units	n	Mean	SD	Median	Min	Max	p-value^a^	Distribution^a^	Method^b^	LRL of RI	URL of RI	CI 90% of LRL	CI 90% of URL
Sodium	mmol/L	243	141	4	141	122	157	<0.00	NG	NP	131	149	122–134	148–156
Chloride	mmol/L	243	108	5	108	94	126	0.11	G	NP	97	118	94–99	116–122
Calcium	mmol/L	275	2.13	0.18	2.15	1.03	2.61	<0.00	NG	NP	1.80	2.41	1.63–1.84	2.4–2.48
Phosphorus	mmol/L	275	2.88	0.65	2.76	1.91	6.37	<0.00	NG	NP	2.08	4.63	1.96–2.12	4.29–5.58
Uric acid	μmol/L	322	0.481	0.228	0.443	0.089	1.389	<0.00	NG	NP	0.160	1.095	0.122–0.180	0.946–1.131
CK	U/L	281	1435	832	1270	314	5873	<0.00	NG	NP	469	3604	438–530	3060–4948
AST	U/L	307	164	47	153	65	370	<0.00	NG	NP	96	301	91–108	281–331
GGT	U/L	57	1	-	1	0	3	<0.00	NG	NP	0	3	-	-
GLDH	U/L	65	3.08	-	3.10	0.30	40.50	<0.00	NG	RT	0.54	16.81	0.39–0.76	11.98–23.29
BOHB	mmol/L	106	0.3	-	0.3	0.0	1.1	<0.00	NG	RT	0.0	1.0	0.0–0.0	0.9–1.1
Amylase	U/L	184	47	27	40	4	156	<0.00	NG	NP	15	130	4.0–18	114–156
Total protein	g/L	319	28	4	28	16	43	<0.00	NG	NP	20	37	17–22	34–39
Albumin	g/L	328	10	2	10	5	21	<0.00	NG	NP	7	14	6.0–8.0	13–16
Globulin	g/L	324	17	2	17	9	27	<0.00	NG	NP	13	22	11.0–14	21–23

RI, reference interval; LRL, lower reference limit; URL, upper reference limit; CK, creatinine kinase; AST, asparate aminotransferase; GGT, gamma glutamyl transferase; GLDH, glutamate dehydrogenase; BOHB, beta-hydroxybutyrate; TS, total solids; TP, total protein

a ^a^Test used for distribution and p-value threshold for interpretation. G, Gaussian; NG, non-Gaussian

b ^b^Statistical method for establishing RI: P, parametric; NP, nonparametric; R, robust; T, transformed

**Table 5:** Age-specific hematologic and blood biochemical reference intervals for wild Carnaby’s cockatoo (*Zanda latirostris)*) nestlings in Western Australia

Analyte	Subgroup	SI Units	n	Mean	SD	Median	Min	Max	*p*-value^a^	Distribution^a^	Method^b^	LRL of RI	URL of RI	CI 90% of LRL	CI 90% of URL
PCV	≤5 weeks	L/L	71	0.34	0.06	0.34	0.20	0.52	0.06	G	R	0.22	0.46	0.20–0.24	0.44–0.48
	6-8 weeks	L/L	169	0.40	0.06	0.40	0.20	0.58	0.00	NG	NP	0.25	0.50	0.20–0.28	0.48–0.58
	≥9 weeks	L/L	75	0.48	-	0.48	0.26	0.62	0.00	NG	RT	0.32	0.59	0.27–0.36	0.57–0.60
Heterophils	≤5 weeks	%	79	63	-	63	11	91	0.02	NG	RT	29	86	21–37	82–89
	6-8 weeks	%	200	54	15	54	18	94	0.07	G	NP	21	78	18–27	76–80
	≥9 weeks	%	93	46	13	45	19	68	0.02	NG	R	20	72	16–24	69–75
Heterophils	≤5 weeks	10^9^/L	79	9.06	-	8.88	1.60	35.60	0.00	NG	RT	2.13	22.88	1.47–3.02	20.23–25.77
	6-8 weeks	10^9^/L	199	8.14	4.65	7.27	0.53	25.27	0.00	NG	NP	1.30	19.42	0.76–1.89	16.28–24.32
	≥9 weeks	10^9^/L	93	6.15	-	6.27	0.80	16.53	0.03	NG	RT	1.44	14.23	1.04–1.95	12.85–15.64
Lymphocytes	≤5 weeks	%	79	30	-	31	6	68	0.00	NG	RT	12	74	11–15	63–87
	6-8 weeks	%	200	40	14	38	5	80	0.07	G	NP	15	70	12–19	67–77
	≥9 weeks	%	93	46	14	45	0	72	0.14	G	R	18	74	14–22	70–78
Lymphocytes	≤5 weeks	10^9^/L	79	4.24	-	4.36	0.92	15.01	0.00	NG	RT	1.30	15.08	1.05–1.60	12.44–17.76
	6-8 weeks	10^9^/L	199	5.64	2.97	5.19	0.70	16.40	0.00	NG	NP	1.10	13.43	0.72–1.62	11.29–16.37
	≥9 weeks	10^9^/L	93	6.40	3.01	6.12	0.00	14.50	0.15	G	R	0.21	12.27	0–1.02	11.27–13.18
Monocytes	≤5 weeks	%	79	3	3	2	0	17	0.00	NG	R	0	9	0–0	6–11
	6-8 weeks	%	200	3	4	2	0	22	0.00	NG	NP	0	16	0–0	12–19
	≥9 weeks	%	93	5	5	3	0	21	0.00	NG	R	0	13	0–0	11–15
Monocytes	≤5 weeks	10^9^/L	79	0.45	0.72	0.21	0.00	3.67	0.00	NG	R	0.00	1.71	0–0	1.23–2.12
	6-8 weeks	10^9^/L	200	0.48	0.77	0.20	0.00	4.66	0.00	NG	NP	0.00	3.38	0–0	2.22–4.17
	≥9 weeks	10^9^/L	93	0.63	0.76	0.34	0.00	4.22	0.00	NG	R	0.00	2.03	0–0	1.53–2.42
															
Total protein	≤5 weeks	g/L	69	26	-	26	16	31	0.00	NG	RT	18	31	15–20	30.21–31.61
	6-8 weeks	g/L	169	29	3	29	17	43	0.00	NG	NP	22	37	17–24	34–43
	≥9 weeks	g/L	80	28	4	27	16	39	0.00	NG	R	19	35	19.00	33.73–36.4
CK	≤5 weeks	U/L	67	1268	-	1263	469	4236	0.00	NG	RT	486	3194	18–21	2697–3777

(Continued)

**Table 5:** Continued

Analyte	Subgroup	SI Units	n	Mean	SD	Median	Min	Max	*p*-value^a^	Distribution^a^	Method^b^	LRL of RI	URL of RI	CI 90% of LRL	CI 90% of URL
	6-8 weeks	U/L	150	1709	889	1577	466	5872	0.00	NG	NP	669	4707	466–731	3331–5873
	≥9 weeks	U/L	63	769	-	741	428	1557	0.00	NG	RT	373	1545	339–412	1335–1748
AST	≤5 weeks	U/L	67	139	33	135	91	293	0.00	NG	R	66	200	45–90	176–221
	6-8 weeks	U/L	167	155	31	150	65	281	0.01	NG	NP	93	220	65–113	208–281
	≥9 weeks	U/L	72	207	59	189	117	370	0.00	NG	R	70	318	50–92	290–345
Amylase	≤5 weeks	U/L	43	60	-	58	18	135	0.01	NG	RT	21	177	17–27	142–213
	6-8 weeks	U/L	100	43	23	38	4	156	0.00	NG	R	0	83	0–4	71–95
	≥9 weeks	U/L	41	37	14	37	14	72	0.54	G	R	8	64	2–14	58–71
Uric acid	≤5 weeks	μmol/L	72	0.465	0.195	0.441	0.122	0.946	0.051	G	R	0.051	0.839	0–0.111	0.761–0.916
	6-8 weeks	μmol/L	171	0.433	0.208	0.378	0.089	1.131	0.000	NG	NP	0.151	1.032	0.089–0.176	0.852–1.131
	≥9 weeks	μmol/L	78	0.553	-	0.534	0.137	1.389	0.001	NG	RT	0.225	1.269	0.197–0.266	1.112–1.487
Sodium	≤5 weeks	mmol/L	61	140	4	140	128	156	0.01	NG	R	131	149	129–133	147–151
	6-8 weeks	mmol/L	131	141	4	141	122	151	0.00	NG	NP	128	148	122–136	147–151
	≥9 weeks	mmol/L	50	145	3	144	139	157	0.00	NG	R	138	151	136–140	149–153
Chloride	≤5 weeks	mmol/L	61	105	5	106	96	120	0.15	G	R	95	115	94–97	113–117
	6-8 weeks	mmol/L	131	107	4	107	94	118	0.07	G	NP	97	117	94–101	116–118
	≥9 weeks	mmol/L	50	112	-	112	104	126	0.04	NG	RT	103	121	101–105	119–123
Albumin	≤5 weeks	g/L	70	10	2	10	5	14	0.00	NG	R	6	13	6–7	12–13
	6-8 weeks	g/L	177	11	2	11	6	21	0.00	NG	NP	8	15	6–8	13–21
	≥9 weeks	g/L	80	10	2	10	6	16	0.00	NG	R	6	13	5–7	13–14
Globulin	≤5 weeks	g/L	70	16	2	16	11	21	0.06	G	R	12	20	11–13	20–21
	6-8 weeks	g/L	174	18	2	18	9	23	0.00	NG	NP	13	22	9.0–15	21–23
	≥9 weeks	g/L	79	18	2	17	10	27	0.00	NG	R	12	22	11–13	21–23
Ca	≤5 weeks	g/L	65	2.13	0.17	2.12	1.78	2.61	0.67	G	R	1.77	2.46	1.71–1.83	2.40–2.53
	6-8 weeks	g/L	149	2.15	0.16	2.17	1.44	2.48	0.00	NG	NP	1.80	2.42	1.44–1.90	2.39–2.48
	≥9 weeks	g/L	60	2.11	-	2.10	1.03	2.40	0.00	NG	RT	1.60	2.37	1.41–1.75	2.33–2.41

RI, reference interval; LRL, lower reference limit; URL, upper reference limit; PCV, packed cell volume; H:L ratio, heterophil to lymphocyte ratio; CK, creatinine kinase; AST, asparate aminotransferase; Ca, calcium

a ^a^Test used for distribution and p-value threshold for interpretation. G, Gaussian; NG, non-Gaussian

b ^b^Statistical method for establishing RI: P, parametric; NP, nonparametric; R, robust; T, transformed

**Table 6:** Results of multiple linear regression analyses of predictor variables for PCV and AST in wild Carnaby’s cockatoo (*Zanda latirostris*) nestlings in Western Australia

	Coefficient (β)	SE	*t*	*p*
PCV				
Age	0.60	0.00	14.09	<0.001
Location	−0.18	0.01	−4.26	<0.001
Hollow type	−0.09	0.01	−2.06	0.04
Chlamydia status	−0.16	0.01	−3.70	<0.001
(Constant)		0.01	19.23	<0.001
Adjusted R^2^	0.43			
F (4, 312) = 61.12, p < 0.001				
AST				
Age	0.80	0.37	7.58	<0.001
Year	−0.12	0.78	−2.47	0.01
Sibling	0.10	5.64	2.07	0.04
Year*age	−0.30	0.08	−2.85	0.01
(Constant)		1566.84	2.49	0.01
Adjusted R^2^	0.33			
F (4, 302) = 38.10, p < 0.001				

BFDV is thought to be endemic in psittacine populations in the eastern states of Australia (Raidal and Peters, 2017; Martens *et al.,* 2020b), however, due to a lack of data, it is not known if this is also the case in the west of the continent. In this study, there was a low proportion of Carnaby's cockatoo nestlings that were PCR positive for BFDV (1.7%; 95% CI, 0.8–3.4). It is not known if this is indicative of a low prevalence among adults in the population, as transmission pathways may be different in adults and nestlings. Nestlings may be more likely to be infected via contaminated nest hollows than by their parents (Eastwood *et al.,* 2019), but adult cockatoos may have increased exposure through contact with infected conspecifics, reservoir hosts, or via contaminated fomites. Moreover, there may be differences in disease prevalence between the geographically isolated breeding populations of Carnaby's cockatoos, such as in the northern and southern parts of their distribution range, or between breeding and non-breeding groups. In contrast, the prevalence of BFDV in other wild cacatuids in Australia has been found to be relatively high; a prevalence of 69.6% (95% CI, 55.2–80.9) was found in wild cacatuids in Victoria (Sutherland *et al.,* 2019) and a prevalence of 45% (95%; CI, 23.1–68.5) was found in sulphur-crested cockatoos (*Cacatua galerita*) (Martens *et al.,* 2020b). A low prevalence of the virus is suggestive of a virus spill-over from reservoir hosts (which may include abundant parrot species, such as galahs (*Eolophus roseicapillus*) and corellas (*Cacatua* spp.), rather than endemic disease. However, ongoing research on the prevalence of infection and subsequent phylogenetic analyses in adult breeding cockatoos will lead to a better understanding of the prevalence of BFDV and its epidemiology in wild Carnaby's cockatoos.

This is the first report of a BFDV genotype identification in a wild Carnaby's cockatoo. This finding constitutes additional evidence of the high BFDV genetic diversity (Sarker *et al.,* 2014b), extends the host range of the virus to include wild Carnaby's cockatoos, and adds to the 24 genotypic variants previously identified in Psittaciformes in Western Australia (Bassami *et al.,* 2001; Sarker *et al.,* 2014a; Sarker *et al.,* 2014b; Das *et al.,* 2016). The novel BFDV genotype identified in this study was most closely related to isolates from red-winged parrots and African grey parrots from Europe, rather than those from Australian Psittaciformes. Although this is interesting, it is important to highlight that the main purpose of the simplified phylogenetic tree presented in this paper was to demonstrate the uniqueness of the sequence recovered, and its likely phylogenetic position. The dataset was not sufficient to represent BFDV deep taxonomy which explains the polytomies observed. Whole genome sequencing of the newly described BFDV is required for a more in-depth phylogenetic representation of its evolutionary relationships with other variants, particularly those found in Australasia, which has been pointed to as the likely geographical origin of this virus (Sarker *et al.,* 2014b). Only then, can inferences about its origin, potential host-switches and mutation rates be made. Again, further sampling of wild Carnaby's cockatoos (including adults) and other Psittaciformes in Australia is essential to achieve a better phylogenetic resolution, as well as to ascertain the prevalence, host-range, geographic distribution, and potential clinical impact of this novel variant on wild Carnaby's cockatoo populations.

APV detected via PCR was also found in a low proportion of Carnaby's cockatoo nestlings (2.9%; 95% CI, 1.7–5.0) in this study. In other Australian parrot species, seroprevalence of APV in various wild parrot species in New South Wales ranged from 0% to 87.5% (Raidal *et al.,* 1998). APV antibody has been detected in a captive Baudin’s cockatoo (*Zanda baudinii*) in Western Australia (Raidal *et al.,* 1998), but otherwise, there are no data on APV in wild cockatoos in Western Australia. Again, further research is required to understand this virus in Western Australian avian species. Five of the 12 Carnaby’s cockatoo nestlings that tested positive for APV (41.7%) had concurrent infections with either BFDV or *Chlamydia*, and three out of seven of the nestlings with BFDV (42.9%) were also infected with APV. Due to the low numbers of birds infected, it is difficult to determine the significance of these co-infections. In other Australian psittacine species, the incidence of infections with BFDV and *Chlamydia* has been found to be statistically independent (Martens *et al.,* 2021).

None of the Carnaby's cockatoo nestlings was seropositive for *C. psittaci* infection, although this was not unexpected given young birds usually do not produce a detectable immune response (Andersen, 1998). Little is known about routes of transmission of *Chlamydia* in wild birds, but nestlings can be infected via regurgitant feeding from their parents, or by inhalation or ingestion of contaminated particles in nest hollows (Brand, 1989). There may be differences in transmission rates based on reproductive activity; in crimson rosellas (*Platycercus elegans*) non-breeding birds are twice as likely to be infected than breeding birds (Stokes *et al.*, 2020b). Previously in this species, *Chlamydia* infection had been detected only in captive Carnaby's cockatoos, at a black cockatoo rehabilitation centre in Perth, Western Australia in 2010. Two Carnaby's cockatoos died from the disease, and a third recovered following treatment, with seropositivity detected in a fourth cockatoo without clinical signs (Le Souëf, 2013). Infection with *C. psittaci* is more common in captive birds, with few data available on prevalence in wild parrots (Stokes *et al.,* 2021a). Studies conducted in eastern Australia have shown a low prevalence of avian chlamydiosis in wild birds (McElnea and Cross, 1999; Amery-Gale *et al.,* 2020). However, a recent study showed higher prevalence in several wild psittacine species and highlighted the many factors that affect detection and prevalence, such as intermittent shedding, seasonal and sex differences (Stokes *et al.,* 2020). Wild birds infected with *Chlamydia* typically show minimal or no direct clinical signs (Andersen and Franson, 2007), but there is little known about the effect of chlamydial infection on wild avian populations, especially at a subclinical level. The pathogen has been detected in free-living blue-fronted Amazon parrots (*Amazona aestiva*) and hyacinth macaws (*Anodorhynchus hyacinthinus*) in Brazil, with the absence of clinical disease suggested by the authors to indicate a stable host–parasite relationship (de Freitas *et al.,* 2006). However, as mentioned previously, these stable relationships can become disturbed when birds are subjected to stress, such as weather changes, nesting, migration and food shortages, and conversion to captivity, precipitating clinical disease (Burkhart and Page, 1971; Mawson, 1997). Mortality events associated with *C. psittaci* infection, presumably caused by changes to the normal latency of infection, are occasionally seen in free-living populations of birds (Mawson, 1997; Andersen and Franson, 2007). More research on the prevalence of *Chlamydia* is needed to determine the significance of this pathogen in Western Australian birds and predict the risk of disease outbreaks. Further investigation into the clinical and subclinical effects of the infection on wild cockatoos would also be informative, given the results of this study which showed a statistical association between *Chlamydia* infection and lower PCV and body condition index. In another study, a negative association with body mass was seen in *C. psittaci* seropositive birds, and *Chlamydiales* positive and seropositive birds had a lower PCV (Stokes *et al.,* 2020).

The cause of the temporal variation in PCR prevalence of pathogens (Table 2) can only be hypothesized. A high number of nestlings (52%) tested positive on PCR for *Chlamydia* infection in 2012, with all positive results originating from Coomallo Creek. Increased shedding of *C. psittaci* in persistently infected birds can occur due to stress (Andersen and Franson, 2007), so a stressor or multiple stressors, such as lower resource availability or increased contact with other avian species, may lead to increased shedding of the bacteria. It may be relevant that a large wildfire destroyed a large area of Carnaby’s cockatoo breeding habitat to the south of the Coomallo Creek site in 2010, and increased aggression among female cockatoos was observed in 2011 and 2012, presumably due to reduced nest hollow resources. It is also important to note that the detection of these temporal fluctuations was only possible due to the long period over which this study occurred. If sampled in only one year, it may have led to the false conclusion that certain pathogens were not present in the populations. This highlights the advantage of longitudinal studies using long-term surveillance, enabling the detection of temporal fluctuations, which is critical for defining and controlling wildlife disease risks (Walton *et al.,* 2016).

No endoparasites were detected in the 120 fecal samples tested during this study. These findings support research on wild black cockatoos admitted to a zoo veterinary hospital for treatment, in which no endoparasites were found in the screening of 33 fecal samples, or in the necropsy examinations of 79 wild Carnaby's cockatoos (Le Souëf, 2013). These findings suggest that endoparasites are rare, or not found in wild Carnaby’s cockatoos, though they are known to be susceptible to infestation with ascarid nematodes (*Ascaridia* sp.) in captivity (Le Souëf *et al.,* 2015), which is likely due to the aviary environment which increases exposure to contaminated soil and feces. Ectoparasites are also rare in wild Carnaby's cockatoos, and it is of potential interest to note that the nestling on which a tick was found during this study had hematological and blood biochemical changes consistent with a stress response (heterophilia and increased CK), which may have led to a higher susceptibility to parasitic infestation.

Age-related variation was found in many of the blood analytes of Carnaby's cockatoo nestlings. In this study, nestlings showed an increase in PCV, RBC, lymphocyte percentage, monocyte percentage, AST, sodium, chloride and globulins with age. Blood parameters that decreased with age were heterophil percentage, H:L ratio and amylase. For total protein, CK, uric acid, albumin and calcium, there was a non-linear variation with age. No significant variation was seen in MCHC, MCH, MCV, eosinophil and basophil counts, BOHB, GGT, GLDH, and phosphorus. Many of these variations are consistent with those seen in other juvenile cockatoos (Clubb *et al.,* 1991). An increase in PCV with age has been found in many psittacines and other wild avian species (Fair *et al.,* 2007; Schwartz and Beaufrere, 2022) and is ascribed to hematopoiesis that occurs during late embryonic growth and the early post-hatching period, leading to an increase in the production of red blood cells by the spleen, liver and bone marrow (Campbell, 1994). In many other psittacine species, WBC counts decrease with age (Karesh *et al.,* 1997; Foldenauer *et al.,* 2007; Vaz *et al.,* 2016), however for the Carnaby's cockatoo nestlings, the WBC did not change significantly, and when compared with the dataset for captive adult Carnaby's cockatoos (Le Souëf *et al.,* 2013), there were no significant differences between adults and nestlings (*p =* 0.07). However, there may be a difference in the leukogram between wild and captive cockatoos, as seen in other species, such as flamingos (Puerta *et al.,* 1992). There was a reduction in the H:L ratio (heterophil percentage increased, and lymphocyte percentage decreased) in Carnaby's cockatoo nestlings, which has been reported in other cockatoos and Eclectus parrots (*Eclectus roratus*) (Clubb *et al.,* 1990; Clubb *et al.,* 1991). In the oldest age group of nestlings, there was a similar percentage of heterophils and lymphocytes. As with most psittacine species, adult Carnaby's cockatoos are heterophilic predominant, so possibly a further shift occurs such that heterophils dominate as the most numerous WBC in mature cockatoos.

The multivariable models for predictors of PCV and AST (Table 6) were relatively strong (adjusted R^2^ = 0.43 and 0.33, respectively) compared with the models for the other analytes. This is likely due to the strong effect of age on PCV and AST, as well as the effect of age; however, there was also a less strong, but still significant effect of location, hollow type, and *Chlamydia* status on PCV. Those nestlings in artificial hollows had a slightly higher PCV than those in natural hollows, and nestlings found at Coomallo Creek had a higher PCV than those found in the other regions. These differences were possibly associated with higher temperatures in artificial hollows, which have been reported, although nestling body condition does not differ between nestlings from artificial and natural hollows (Saunders *et al.,* 2020). These results indicate that environmental factors may affect the blood analytes of nestlings and should be considered when interpreting blood results. Changes in hematologic values associated with changes in environmental temperatures, and state of nutrition are often seen in wild birds (Fair *et al.,* 2007; Schwartz and Beaufrere, 2022).

Previous findings in Carnaby's cockatoos have shown that sibling pairs would usually both show body mass within the benchmark ranges (Saunders *et al.,* 2014). When sibling pairs were analysed in this study, there was a significantly higher CK measurement in younger siblings, which may indicate a higher level of stress experienced by these nestlings. Otherwise, there was no significant effect of hatching order on blood analytes and body condition index, indicating that younger siblings, at least by the age of 23 days, the age of the youngest sibling in the dataset, are likely to be no less clinically healthy than older siblings. This is in contrast with studies in other species which show less healthy blood profiles and body condition scores in last-born nestlings or between broods (Norte *et al.,* 2008; Neb *et al.,* 2019; Zaremba *et al.,* 2021).

Baseline health data on extant wildlife populations provide valuable information on reference interval data and pathogen loads in a particular species (Mathews *et al.,* 2006). This study contributes baseline health data of nestlings to the current knowledge of wild Carnaby's cockatoo populations and includes disease prevalence data as well as reference intervals for blood hematology and biochemistry, which will aid in ongoing surveillance and conservation management for this endangered species. The data also fill a long-standing knowledge gap in the recovery plan for this species.

## Supplementary Material

Web_Material_coae005

## Data Availability

Data available on request.
