# Supplementary material for: Health parameters for wild Carnaby's cockatoo (Zanda latirostris) nestlings in Western Australia: results of a long-term study
Source: Conserv Physiol. 2024 Feb 20;12(1):coae005. doi: 10.1093/conphys/coae005 (PMC11784592; doi:10.1093/conphys/coae005)
Supplement: Web_Material_coae005 [file web_material_coae005.pdf]

SUPPLEMENTARY MATERIAL

Table 1. Results of multiple linear regression analyses of predictor variables for blood analytes in wild Carnaby's cockatoo (*Zanda latirostris*) nestlings in Western Australia

	Coefficient (β)	SE	*t*	*p*
WBC				
Age	-0.10	0.03	-1.97	0.05
Location	-0.10	0.53	-2.07	0.04
Hollow type	-0.17	0.70	-3.26	0.001
Sex	0.16	0.68	3.14	0.002
(Constant)		1.34	13.13	<0.001
Adjusted R^2	0.06			
$F (4, 367) = 6.49, p < 0.001$				
Absolute heterophil count				
Age	-0.22	0.02	-4.42	<0.001
Sex	0.12	0.47	2.35	0.02
(Constant)		0.88	12.54	<0.001
Adjusted R^2	0.06			
$F (2, 371) = 12.45, p < 0.001$				
Absolute lymphocyte count				
Age	0.11	0.01	2.13	0.03
Location	-0.14	0.23	-2.76	0.01
Hollow type	-0.22	0.30	-4.40	<0.001
Sex	0.15	0.30	2.94	0.01
(Constant)		0.59	8.36	<0.001
Adjusted R^2	0.09			
$F (4, 370) = 9.81, p < 0.001$				
Absolute eosinophil count				
Location	-0.11	0.02	-2.18	0.03
Hollow type	-0.10	0.03	-2.00	0.046
(Constant)		0.02	12.40	<0.001
Adjusted R^2	0.02			
$F(2, 373) = 4.25, p =0.02$				
H:L Ratio				
Age	-0.24	0.01	-4.74	<0.001
Sibling	0.13	0.21	2.59	0.01
(Constant)		0.30	10.11	<0.001
Adjusted R^2	0.07			
$F (2, 365) = 14.61, p < 0.001$				
Total protein				
Age	0.16	0.02	3.04	<0.001
Location	-0.14	0.32	-2.51	0.01
Sibling	-0.13	0.52	-2.40	0.02
(Constant)		0.77	34.26	<0.001
Adjusted R^2	0.06			
$F (3, 320) = 7.312, p < 0.001$				

CK				
Age	-0.26	3.56	-4.43	<0.001
Year	-0.15	21.50	-2.52	0.01
(Constant)		200.34	11.91	<0.001
Adjusted R^2	0.07			
$F(2, 281) = 12.07, p < 0.001$				
Uric acid				
Age	0.23	0.00	4.32	<0.001
Location	-0.12	0.02	-2.25	0.03
Hollow type	-0.20	0.02	-3.82	<0.001
(Constant)		0.05	7.60	<0.001
Adjusted R^2	0.10			
$F(3, 323) = 13.42, p < 0.001$				
Globulin				
Age	0.20	0.01	3.69	<0.001
Location	-0.13	0.18	-2.40	0.02
Sibling	-0.13	0.30	-2.37	0.02
Body condition index	-0.13	0.01	-2.48	0.01
(Constant)		0.44	36.40	<0.001
Adjusted R^2	0.09			
$F(4, 324) = 8.78, p < 0.001$				
